# Laser Powder Bed Fusion Additive Manufacturing of a CoCrFeNiCu High-Entropy Alloy: Processability, Microstructural Insights, and (In Situ) Mechanical Behavior

**DOI:** 10.3390/ma18133071

**Published:** 2025-06-27

**Authors:** Vito Burgio, Ghazal Moeini

**Affiliations:** Institute of Mechanical Engineering, Westphalian University of Applied Sciences, Neidenburger Straße 43, 45897 Gelsenkirchen, Germany; ghazal.moeini@w-hs.de

**Keywords:** additive manufacturing, high-entropy alloys, powder bed fusion, microstructure, mechanical properties

## Abstract

High-entropy alloys are known for their promising mechanical properties, wear and corrosion resistance, which are maintained across a wide range of temperatures. In this study, a CoCrFeNiCu-based high-entropy alloy, distinguished from conventional CoCrFeNi systems by the addition of Cu, which is known to enhance toughness and wear resistance, was investigated to better understand the effects of compositional modification on processability and performance. The influence of key process parameters, specifically laser power and scan speed, on the processability of CoCrFeNiCu-based high-entropy alloys produced by laser powder bed fusion additive manufacturing was investigated, with a focus of low laser power, which is critical for minimizing defects and improving the resulting microstructure and mechanical performance. The printed sample density gradually increases with higher volumetric energy density, achieving densities exceeding 99.0%. However, at higher energy densities, the samples exhibit susceptibility to hot cracking, an issue that cannot be mitigated by adjusting the process parameters. Mechanical properties under optimized parameters were further evaluated using Charpy impact and (in situ) tensile tests. These evaluations were supplemented by in situ tensile experiments conducted within a scanning electron microscope to gain insights into the behavior of defects, such as hot cracks, during tensile testing. Despite the sensitivity to hot cracking, the samples exhibited a respectable ultimate tensile strength of 662 MPa, comparable to fine-grained steels like S500MC (070XLK). These findings underscore the potential of CoCrFeNiCu-based high-entropy alloys for advanced applications. However, they also highlight the necessity for developing strategies to ensure stable and reliable processing methods that can mitigate the susceptibility to hot cracking.

## 1. Introduction

High-entropy alloys (HEA) are defined as alloys containing at least five principal elements, each with an atomic percentage between 5 and 35% [1]. Additionally, they are defined as alloys having configurational entropies at a random state larger than 1.5R, independent of their phase composition at room temperature [1]. CoCrFeNi-based alloys are also often considered as HEA in literature since their composition and configurational entropy are close to the lower limits of both definitions [1].

HEAs have better mechanical properties such as high temperature strength, fatigue properties, ductility, and corrosion resistance compared to traditional alloys [2].

Among the family of high-entropy alloys, CoCrFeNi-HEAs prefer to form a stable single FCC phase, thus serving as a base alloy in HEA design to achieve comprehensive properties [3].

Due to face-centered cubic (FCC) lattice structure and combination of several strengthening mechanisms, such as solid solution strengthening, Hall–Petch effect, and precipitation strengthening, this group of HEAs exhibits excellent ductility with good mechanical properties in a huge temperature range from ambient temperatures up to cryogenic temperatures [4].

A common method is to alloy with other elements, enabling solid solution and/or interstitial strengthening mechanisms [5]. Additionally, the introduction of nanoparticles into the base matrix further enhances strength through precipitation strengthening [5]. Introduction of copper (Cu) and manganese (Mn) into the alloy offers several advantages.

Mn is an FCC phase-forming element; by increasing its content, the volume fraction of the FCC phase is increased in high-entropy alloys. This increase has an impact on the mechanical properties, such as compressibility and wear resistance, and eventually on the corrosion resistance [6].

Cu serves as a key element for precipitation-hardening in steels due to its limited solubility in iron-based systems. An example of this is the Super 304H austenitic heat-resistant steel, which is a modified version of 304 stainless steel with an addition of 3 wt% copper. This composition significantly enhances its performance at elevated temperatures, primarily due to the precipitation strengthening effect of nanoscale, copper-rich phases within the FCC-matrix [7]. Studies have shown that the addition of Cu enhances the ductility of HEAs [8]. As a result, copper tends to segregate and therefore promotes the formation of Cu-rich precipitate phases that contribute to the alloy’s improved ductility.

Manufacturing processes have a strong influence on the mechanical properties of HEAs. It has been determined that the CoCrFeNi part produced by the hot-rolling process has a better yield and ultimate strength compared to the casted parts [9,10,11,12]. Additive manufacturing processes, such as Laser-Powder Bed Fusion (L-PBF), can improve the mechanical properties of high-entropy alloys due to their inherent unique processing characteristics. Specifically, the rapid solidification and repeated remelting associated with these techniques facilitate the formation of fine grains and numerous substructures with a high density of dislocations, thereby enhancing the yield strength compared to conventionally processed alloys. Brif et al. [13] showed that the yield strength of the CoCrFeNi alloy produced by the powder bed fusion (PBF) process is twice, respectively, the treble of that alloy in as-cast condition, and Kuzminova [9] showed in turn that the yield strength of alloys using L-PBF is twice than that of hot-rolled alloys. However, the thermal cycling and partial remelting experienced by previous layers can significantly increase the hot cracking susceptibility of superalloys [14].

Recent advances in high-entropy alloy research have leveraged thermal analysis, phase prediction, and mechanical behavior characterization—often using machine learning and computational thermodynamics—to deepen understanding of phase stability, microstructural evolution, and mechanical response in complex HEA systems [15,16,17,18]. These approaches enable more accurate prediction of phase formation and mechanical properties, guiding alloy design and reducing experimental trial and error. However, the specific effects of combining Cu and Mn with CoCrFeNi, particularly under the rapid solidification and thermal cycling of additive manufacturing, remain insufficiently explored.

Sun et al. reported extensively about the hot-cracking mechanisms during additive manufacturing of CoCrFeNi [19]. They reported intergranular hot cracks and high residual stresses in the near vicinity of these cracks. They also showed that hot-cracking susceptibility increases with large, columnar grains and described this behavior with Rappaz-Drezet-Gremaud criterion. However, their alloy showed no segregations at high-angle grain boundaries, which also favors hot-crack formation by maintaining the liquid film at the end of the solidification.

In another study, Niu et al. conducted a parameter study with CoCrFeNi-HEA with Mn additions [20]. They could not find a parameter without hot cracking and concluded that the alloy is unsuitable for the L-PBF process. This is mainly attributed to the rapid solidification, resulting in high stress concentrations during the L-PBF process. In addition, large columnar grains favor hot cracking due to the rigidity of strains and long intergranular liquid channels [21]. It is reported that fine precipitates (or segregations) of aluminum at grain boundaries can help to suppress cracks in CoCrFeNi-HEAs [22,23].

While substantial research has explored the L-PBF of high-entropy alloys, this work on CoCrFeNiCu addresses a critical gap by focusing on hot-cracking susceptibility at relatively low laser powers, a parameter space that has received limited attention in prior studies, which often prioritize density or mechanical properties without deeply investigating crack formation mechanisms. In contrast, our study on CoCrFeNiCu HEAs processed by L-PBF also observed hot cracking that could not be mitigated by process parameter adjustment, but the addition of Cu may influence the solidification behavior and residual stress distribution differently than in the equimolar CoCrFeNi system [19]. Furthermore, while previous work has shown that cracks are often located at high-angle grain boundaries and are linked to regions of high mechanical solicitation during solidification [24], our in situ mechanical testing provides new insights into how these cracks behave under load, which has not been extensively reported for Cu-containing HEAs.

In this context, the laser power P and scan speed v were the variable parameters, systematically varied to evaluate their influence on the microstructure and mechanical properties (including density, hardness, tensile strength, and impact toughness), enabling the assessment of how modifications in process parameters affect these properties. To investigate the processability and hot cracking susceptibility, the study employs a 3D printer equipped with a relatively low laser power (up to 250 W) compared to most of the available studies that use higher laser power (>250 W) for fabrication of the additively manufactured HEA parts [25,26].

A batch of tensile specimens was tempered to reduce residual stress [27] resulting from the printing process, which is typical of additively manufactured parts [28]. Heat treatment was conducted at 400 °C for 2.5 h aimed at achieving stress relief and subtle microstructural adjustments without triggering significant phase transformations or coarsening. Studies on similar HEAs show that annealing at low to moderate temperatures (300–500 °C) can reduce internal stresses and dislocation density but may also slightly decrease strength or ductility due to recovery processes, while avoiding the pronounced softening, grain growth, or phase separation that occur at higher temperatures (above 700–800 °C) [29]. In CoCrFeNiCu systems, higher temperature treatments (e.g., 800–1100 °C) can lead to the precipitation and coarsening of Cu-rich phases, transformation between FCC phases, and a general reduction in hardness and thermal stability due to microstructural coarsening and phase changes [30].

Furthermore, in situ tensile testing using a scanning electron microscope (SEM) was conducted to characterize the deformation behavior and crack propagation mechanisms at the microscale.

## 2. Materials and Methods

### 2.1. Powder Material

Argon atomized equiatomic pre-alloyed CoCrFeNi powder (m4p material solutions GmbH, Magdeburg, Germany), with copper (Cu) and manganese (Mn) additions was used. The chemical composition is summarized in Table 1. For simplicity and clarity, this alloy will hereafter be referred to as CoCrFeNiCu.

**Figure 1:**
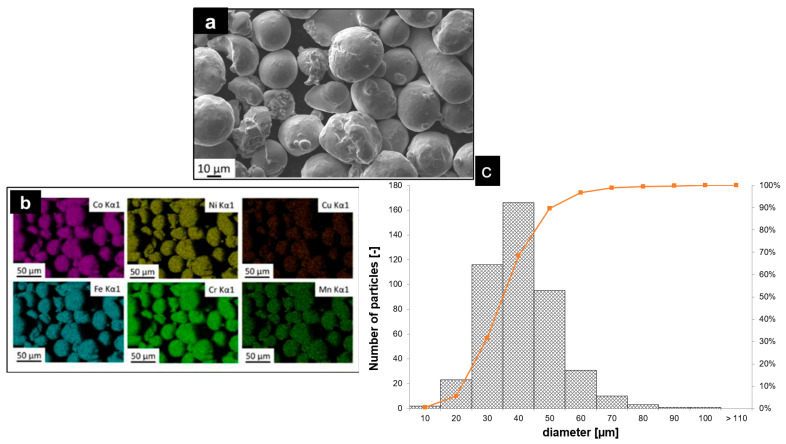
(**a**) SEM micrograph showing the powder morphology; (**b**) EDS maps of elements distribution; (**c**) particle size distribution.

Image analysis was performed to measure particle diameter distribution and assess deviations from the ideal spherical shape of the powder. The results revealed a predominantly spherical particle morphology, which facilitates the formation of dense, uniform powder bed layers. However, some satellites and agglomerates were observed. The particle size distribution, plotted in Figure 1c, follows a lognormal distribution, with diameters ranging from 15 to 45 μm and a mean value of 36.3 μm ± 11.5 μm. As shown in Figure 1b, energy-dispersive X-ray spectroscopy (EDX) mapping indicates a homogeneous element distribution, with no evidence of significant segregation. A summary of these powder characterization results is provided in Figure 1.

### 2.2. Sample Manufacturing and Testing

Additive manufactured specimens were fabricated using the OR LASER Orlas Creator SLM 250 system (formerly O.R. Lasertechnologie GmbH, Dieburg, Germany). This system is equipped with a 1070 nm Yb fiber laser with a beam diameter of 40 μm, a maximum laser power of 250 W, and a rotating rubber coater blade. During the manufacturing process, nitrogen was injected into the build chamber as shielding gas. A design of experiments (DoE) approach was employed to systematically identify optimal process parameters. This approach ensured efficient use of resources while addressing machine-caused constraints. First, SSTs are visually inspected with optical microscopy, followed by the fabrication of density cubes, according to recommendations of VDI 3405 Part 2 [31].

Once suitable process parameters were identified, firstly cubes of 10 × 10 × 10 [mm] size and afterwards specimens for impact testing and (in situ) tensile tests were produced. Laser power (P) and scan speed (v) were the variable parameters, whereas layer thickness (t) and hatching distance (h_s_) were kept constant.

To show the influence of process parameters in terms of the volume energy density (VED) on density and microstructure, VED [J/mm^3^] can be calculated using the following equation [31]:

$$\mathrm{VED}=\frac{P_{L}}{v\;*\;h_{s}*\;t}$$

Neither the powder nor the build platform was pre-heated to simplify the process and assess the baseline behavior. A central composite design (CCD) was employed to combine the process parameters systematically. SST experiments were conducted to observe the interaction between the laser and the material, varying laser power between 52 and 250 W and scan speed between 200 and 1500 mm/s. As mentioned before, after narrowing down the process window, nine density cubes (10 × 10 × 10 [mm]) were fabricated with a layer thickness of 25 µm and a hatching distance of 80 µm. The scanning direction was set at the rotation angle of 45° in a zigzag pattern between the consecutive layers. Three of the six as-fabricated samples were heat treated at 400 °C for 2.5 h in a muffle fur-nace (Nabertherm LT 9/14, Nabertherm GmbH, Lilienthal, Germany) aimed at achieving stress relief and subtle microstructural adjustments without triggering significant phase transformations or coarsening.Microstructural observations were performed on cross-sections using a scanning electron microscope (Zeiss Sigma 300 VP, Carl Zeiss Microscopy Deutschland GmbH, Oberkochen, Germany) equipped with an energy-dispersive spectrometer (EDS). Energy dispersive X-ray spectroscopy (EDX) analysis was performed using a Zeiss Sigma 300 VP field emission gun scanning electron microscope (FEG-SEM) at an accelerating voltage of 20 kV and a working distance of 8.5 mm. The crystal orientation was observed by electron backscattered diffraction (EBSD) as shown by scanning electron microscopy (C NANO). Electron backscattered diffraction (EBSD) analysis was performed using an accelerating voltage of 20 kV, a working distance of 18.5 mm, and a 0.15 µm step size for investigating an area of 1085 µm × 814 µm along the build direction.

Quantitative analysis of stitched images captured by an optical microscope (OM) was performed using digital image processing. The images were binarized, classifying each pixel as either material or pore.

**Figure 2:**
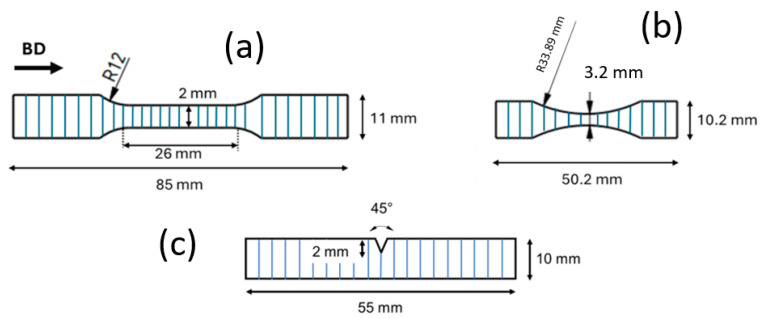
Geometry of (**a**) tensile specimen according to DIN 50125, Form E; (**b**) miniaturized tensile test specimen for in situ testing; (**a**,**b**) specimens having a thickness of 2 mm; (**c**) Charpy impact test specimen according to DIN EN ISO 148-1 [33].

Microhardness testing was carried out using a KB30S automatic micro/macro hardness testing machine (KB Prüftechnik GmbH, Hochdorf-Assenheim, Germany) with a load of 1000 g and a dwell time of 13 s. Following metallographic investigation and hardness testing, six tensile specimens were fabricated in accordance with DIN 50125, Form E (see Figure 2a). Tensile tests were conducted at room temperature following DIN EN ISO 6892-1 [32] (Method B) using a Zwick/Roell Z100 universal testing machine (Ulm, Germany) at a strain rate of 2.8 × 10^−4^ s^−1^. Additionally, four Charpy impact test specimens (see Figure 2c) were tested at room temperature as well as at −40 °C, according to DIN EN ISO 148-1 [33].

For in situ investigations in the SEM, micro-tensile tests were conducted using a K&W micro-tensile testing module (Fmax = 5 kN, Kammrath & Weiss GmbH, Schwerte, Germany). The sample geometry for the micro-tensile specimens is presented in Figure 2b. Prior to testing, micro-tensile samples were ground on both sides and polished to 1 µm on the observation surface, as described previously. During in situ testing, experiments were conducted with a speed of 10 µm/s and interrupted several times to capture the deformation mechanisms in SEM.

## 3. Results

### 3.1. L-PBF Processability

#### 3.1.1. Single Scan Tracks (SST)

Given the wide range of possible factor combinations, first of all, two sets of nine single-track experiments were conducted to identify a viable process window. Optical microscopy of the SSTs highlighted that low energy input causes lack of fusion, resulting in a non-continuous melt track, whereas too high energy input causes balling.

**Figure 3:**
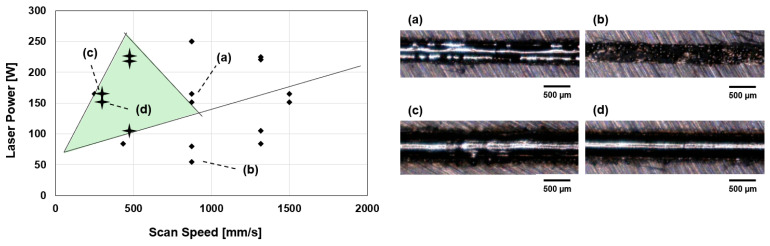
Parameter window of SSTs and the reduced process parameter window obtained using the results from the OM analysis of the SSTs. (**a**) SST with balling; (**b**) SST with no powder melted; (**c**) homogeneous SST with near keyholing; (**d**) homogeneous SST with no visible defect.

Higher scan speeds (>>875 mm/s) combined with high laser power (>>150 W) resulted in defects such as balling (Figure 3a). Energy inputs below 80 W showed virtually no melting of the metal powder, presented in Figure 3b. Laser powers between 100–225 W and scan speeds of 250–500 mm/s resulted in continuous and relatively homogeneous melt tracks (Figure 3c,d). However, the melt track in Figure 3c shows the start of inhomogeneity in the form of accumulating material. Apparently, (P = 150 W, v = 200 mm/s), this parameter set almost leads to keyholing. Figure 3d shows a homogeneous SST without any visible defect. In accordance with these results, a reduced parameter window with laser power between 84–225 W and scan speed between 250–433 mm/s is marked with a green triangle in Figure 3 and is used for the manufacturing of density cubes.

#### 3.1.2. Investigation of Density Cubes

A wide set of process parameters was investigated to obtain suitable parameters for the 3D printer in use. As mentioned before, the other process parameters such as laser power and scan speed are varied to obtain optimized parameters. Optical microscopy of the SSTs highlighted that low energy input causes lack of fusion, resulting in a non-continuous melt track, whereas too high energy input causes balling, refer to Figure 3. Due to this, laser power was kept between 84–225 W and scan speed between 277–433 mm/s to manufacture the density cubes.

**Figure 4:**
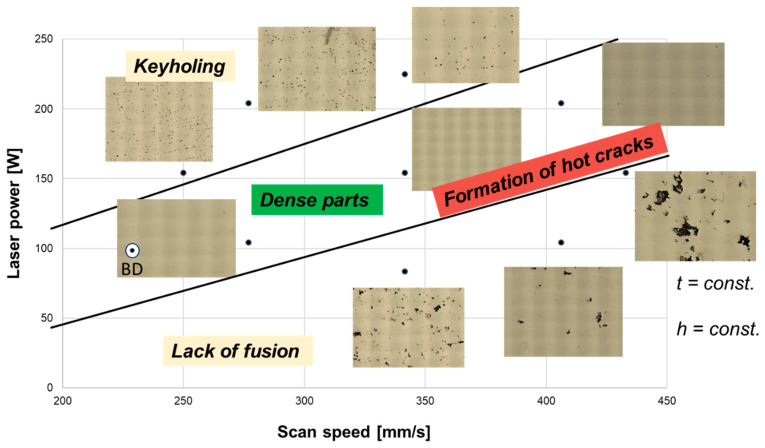
Effect of process parameters (laser power and scan speed) on defect distribution of the L-PBF processed CoCrFeNiCu.

Figure 4 shows the dependence of defect distribution on the processing parameters in the L-PBF-processed CoCrFeNiCu-HEA. All density cubes contain a considerable number of cracks, which tend to increase with increased energy density.

Cracks were detected in all specimens. Additionally, lack of fusion and insufficient melting was identified for VED less than 178 J/mm^3^. Observations showed that the formation of the cracks increased when the VED was more than 225 J/mm^3^. However, keyholing was identified for VED > 300 J/mm^3^. Density cubes manufactured with the parameter set with P = 107 W, v = 277 mm/s, h_s_ = 80 μm, and t = 25 μm showed the highest density (99.8%) and simultaneously the lowest occurrence of cracks. Therefore, all samples for Charpy impact tests and (in situ) tensile tests in Section 3.3 are manufactured with this parameter set.

### 3.2. Microstructure and Crack Morphology

**Figure 5:**
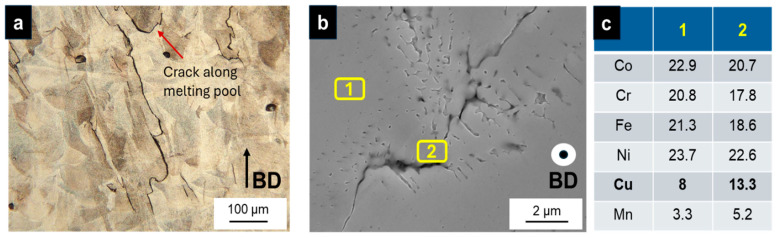
(**a**) OM micrograph of the etched microstructure; (**b**) SEM micrograph of dendritic structures and (micro-)cracks with locations of EDS Measurements; (**c**) EDS measurements (wt%).

The complex temperature cycle in L-PBF with a rapid cooling rate can cause thermal residual stress in the solidification substructure, which induces cracks. To further investigate the characteristics of the printed sample, the microstructure was characterized by SEM and EDX analyses, as shown in Figure 5a,b. Intergranular and transgranular cracks were observed in the printed sample.

EDX analysis showed furthermore that the cracks were irregular, and their size was in the range from tens to hundreds of micrometers (Figure 5a,b).

Quantitative analysis of the SEM images revealed that the average crack density decreased from 12.3 cracks/mm^2^ at a VED of 251.2 J/mm^3^ to 4.3 cracks/mm^2^ at a VED of 187.7 J/mm^3^, while the maximum observed crack length also decreased from 193.3 μm to 84.2 μm over the same range. This trend demonstrates a clear inverse relationship between VED and both crack density and crack length, consistent with findings in the literature that higher energy input can increase thermal gradients and residual stresses, thereby promoting crack formation, while lower VEDs may reduce cracking but risk lack of fusion or porosity.

SEM investigations near (macro-) cracks, presented in Figure 5b, revealed dendritic structures and (micro-) cracks. EDX analysis (Figure 5c) identified significant segregation and precipitation of Cu and Mn in these regions. The approximate measurement locations are marked in Figure 5b, where (1) indicates an area without visible dendritic structures and (2) corresponds to a region near a micro-crack.

**Figure 6:**
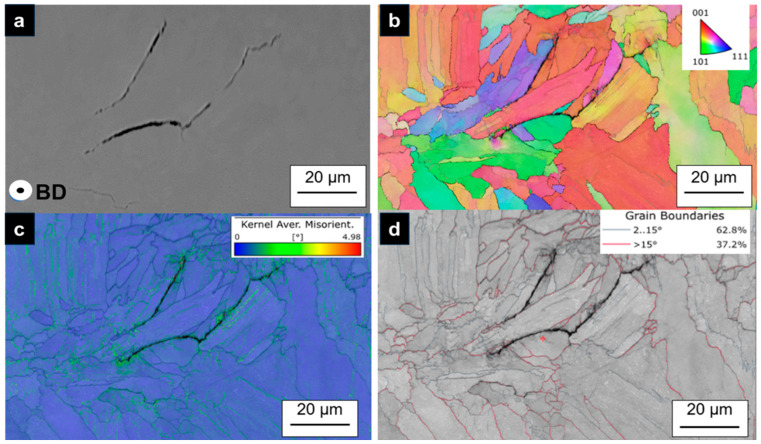
(**a**) SEM micrographs showing a crack at a triple junction of several adjacent grains, (**b**) inverse pole figure (IPF) color map showing cracks and grain structure. (**c**) Kernel average misorientation (KAM) map of the sample and (**d**) EBSD image quality map. (For interpretation of the references to color in this figure legend, the reader is referred to the web version of this article).

EBSD analysis revealed that several cracks formed predominantly along high-angle grain boundaries (HAGBs) in which the grains on both sides of the boundary have different orientations, as shown in Figure 6b. All cracks are distributed along grain boundaries for both columnar and equiaxed grains. Kernel average misorientation (KAM) distribution maps in Figure 6c indicated that the cracking sites displayed much higher KAM values than the other regions.

### 3.3. Mechanical Testing

#### 3.3.1. Hardness Testing

Density cubes with more than 99.0% density and without any crucial imperfections were subjected to hardness testing. For the approach to hardness mapping, 5 rows × 35 measurements per sample were applied. The highest hardness (254 HV1) was measured on the cube with almost the lowest VED of 187.7 J/mm^3^ and highest density (99.8%) and lowest occurrence of cracks. With increasing input energy, the porosity impact also increases, which leads to hardness decreasing from 241 HV1 (220 J/mm^3^) to 225 HV1 (250 J/mm^3^).

Additionally, the most regular distribution of hardness values was observed on the cube manufactured with the lowest VED (187.7 J/mm^3^), resulting from the lowest defect density.

#### 3.3.2. Charpy Impact Testing

The results of Charpy impact tests are presented in Table 2. The impact energies for as-built specimens range from 40 J (for vertically built) to 29 J (for horizontally built samples), representing the highest and lowest values observed. Heat-treated samples exhibit impact energies of 31 J for vertically built and 33 J for horizontally built specimens. Lowering the testing temperature to −40 °C shows no significant change in the impact energy for either vertically (34 J) or horizontally (29 J) built heat-treated specimens.

#### 3.3.3. Tensile Behavior

Table 3 shows the results of tensile testing of the as-printed (AS) and heat-treated (HT) specimens.

Both heat-treated (HT) and as-built (AS) samples exhibit a uniform ultimate tensile strength (UTS) with relatively low variation (662 MPa ± 14). In contrast, yield strength (YS) shows a difference: heat-treated samples demonstrate higher YS with lower variation (541 MPa ± 11.1) compared to as-built samples (496 MPa ± 26). Elongation at break (A) varies widely between 12% and 29%, showing no clear dependence on the heat treatment conditions.

Nevertheless, more investigations on the effect of heat treatment on the mechanical properties on additively manufactured CoCrFeNiCu alloy are needed.

#### 3.3.4. In Situ Tensile Testing

In situ tensile tests were conducted using a scanning electron microscope (SEM) equipped with a micro-tensile machine. The tensile rate was 10 µm/s, and the gauge dimensions of the in situ tensile samples were 2.0 mm (length) × 3.2 mm (width) × 2.0 mm (thickness). The sample was loaded up to F_max_ = 5100 N, without reaching fracture.

**Figure 7:**
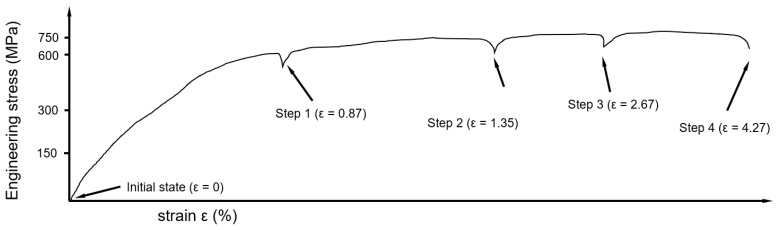
Exemplary engineering stress–strain curve measured during the in situ tensile test, showing the different points of interruption.

The exemplary engineering stress–strain curve, with the interruptions at the regions of interest (ROIs), is shown in Figure 7.

Damage progression was monitored across three ROIs over four different load steps.

**Figure 8:**
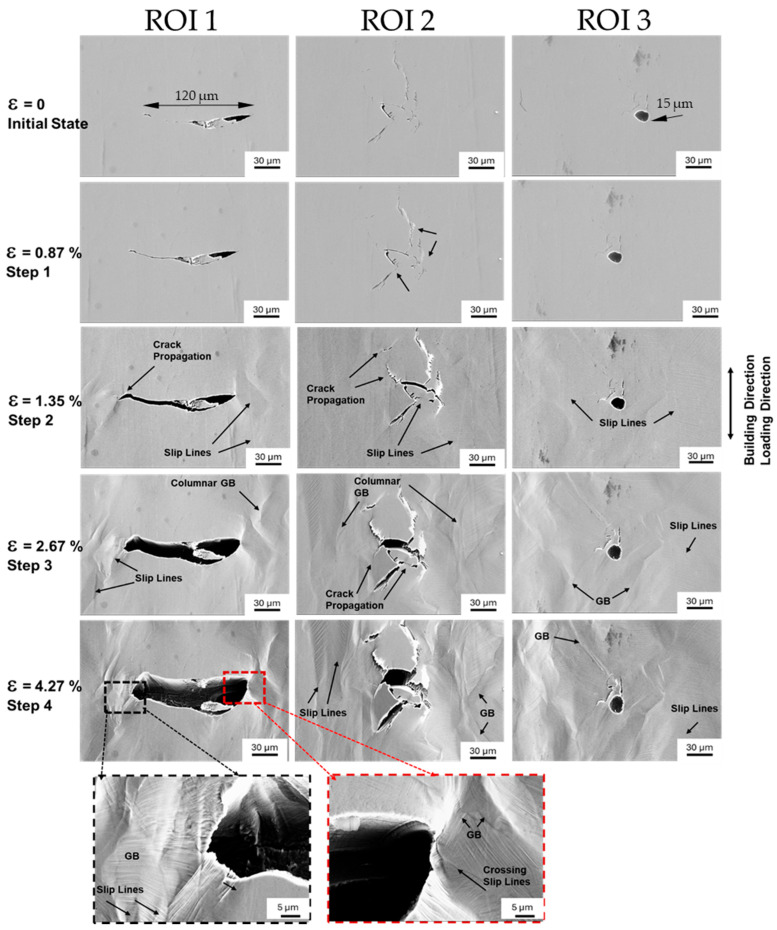
Secondary electron micrographs recorded before reaching the UTS.

The first ROI—ROI 1—was a crack approximately 120 µm long, oriented perpendicular to the loading direction and build direction (BD). The second ROI—ROI 2—comprised a network of cracks, consisting of multiple cracks parallel to BD and with an angle of ca. 45° to BD. The third ROI—ROI 3—was a circular pore 15 µm wide, with two cracks extending in the loading direction above the pore. The initial states of all three ROIs, without tensile loading, are shown in Figure 8 (initial state).

The first interruption of the tensile loading was at a stress of σ = 575 MPa and displacement of ε = 0.87%. At this stress level, seen in Figure 8 (Step 1), for ROI 1, the slight opening of the crack was observed. In this load step, the crack network shows either opening of a crack or merging of two cracks, as indicated by the lower arrow in Figure 8 (Step 1, middle). Figure 8 (Step 1, right) displays the pore with associated cracks; however, perceived changes in contrast compared to the initial state are negligible.

The second interruption of the tensile loading was at σ = 650 MPa and ε = 1.35%, labeled as “Step 2” in Figure 8, marking the transition to the plastic region. Figure 8 (Step 2) presents the damage progression at the aforementioned ROIs. The perpendicular crack to the loading direction (Step 2, ROI 1) appeared to open further, with the left tip propagating several micrometers. At this load level, slip lines and grain boundaries are visible at both tips of the crack, indicating the formation of a “plastic zone.” In the crack network (Figure 8, Step 2, ROI 2), openings and propagation of previously unopened cracks at ca. ±45° to BD were observed, as highlighted by arrows. Slip lines materialized between two cracks in the middle of ROI 2, emphasized by a longer arrow. Surface topography changes were also apparent in the SEM image’s right section. Figure 8 (Step 2, ROI 3) shows the pore with two cracks in BD; although both cracks are oriented in the loading direction, they only showed slight opening. The pore appeared slightly rounder, with slip line formation and emerging grain boundaries visible.

The third interruption during tensile testing occurred at a stress of σ = 715 MPa and a displacement of ε = 2.67%, marked as “Step 3” in Figure 8. The perpendicular crack (Step 3, ROI 1) opened up to approximately 10 µm, with a particle in the crack almost separating from the upper edge. Both regions at the right and left sides of the crack seem more plastically deformed than the regions above or below. Figure 8 (Step 3, ROI 2) illustrates the damage evolution in the crack network. On the left side of the image, a columnar grain is observed, displaying distinct slip lines on both sides of its grain boundaries. Cracks on either side of the network appear brighter, indicating a further change in surface topography with increasing load. A new ca. 15 µm long crack is observed propagating from the middle toward the lower left side of the image, marked with an arrow. Another new crack is visible in the central region of this ROI, which was previously characterized by distinct slip lines between two cracks. This area is marked with the arrow on the right side of the label “crack propagation.” Figure 8 (Step 3, ROI 3) illustrates the damage evolution around the pore, which now appears more oval-shaped and distorted in the loading direction. The crack on the left side of the pore seems to have slightly propagated and deformed along the observation direction. On the right side of the pore, a columnar grain with nearly parallel grain boundaries and visible slip lines became visible. Further to the right, more pronounced slip lines are evident, indicating localized deformation in this region.

The final interruption during tensile loading occurred at a stress of σ = 733 MPa and a displacement of ε = 4.27%, as marked by “Step 4.” The perpendicular crack (Step 4, ROI 1) widened to about 30 µm, with the particle fully detaching from the crack’s upper edge. Damage intensity increased at both crack ends, whereas minimal deformation was noted on the upper and lower sides. In the higher magnification of the crack’s left tip, pronounced crossing slip lines were visible. The right tip’s upper region, at higher magnification, displayed two pronounced grain boundaries 5 µm apart, with visible crossing slip lines. Figure 8 (Step 4, ROI 2) shows most cracks interconnected, with a roughened surface and more prominent grain boundaries. Slip lines in the columnar grain on the left intersected. The middle region of the crack network appeared loosely connected, suggesting potential weakening. In the ROI with the pore and cracks in BD (Figure 8, Step 4, ROI 3), the initially circular pore elongated by approximately 8 µm along the loading direction, and potential crack initiation sites were brighter.

In conclusion, the in situ tensile tests revealed significant damage evolution within the selected ROIs. The observations highlight the transition from elastic to plastic deformation and the localized microstructural changes associated with slip line formation and grain boundary visibility. Despite these pronounced changes, crack propagation demonstrated stability across the loading conditions.

## 4. Discussion

### 4.1. Hot Cracking Mechanism

Our results demonstrate the feasibility of manufacturing CoCrFeNiCu using the L-PBF process. However, the hot-cracking susceptibility of this alloy remains a significant challenge to its widespread application. The crack formation mechanism observed in this study is identified as hot cracking, which is supported by three key findings: (1) dendritic structures observed beneath the crack surface, (2) the segregation of Cu and Mn—both low-melting-point elements—in the vicinity of the cracks, and (3) intergranular cracks identified through EBSD measurements, which revealed high residual stress concentrations at high-angle grain boundaries (HAGBs). These observations suggest that solid-state crack formation mechanisms can be excluded [34].

The precise nature of the hot-cracking mechanism, whether it be solidification or liquation cracking, remains ambiguous. Compared to previous studies, our findings both align with and extend the current understanding of hot-cracking in HEAs. Notably, Sun et al. [19] reported that in additively manufactured CoCrFeNi alloys, intergranular hot cracks are primarily associated with large columnar grains and high residual stresses, with minimal elemental segregation at grain boundaries. In contrast, our study on CoCrFeNiCu reveals pronounced segregation of Cu and Mn at HAGBs, suggesting the persistence of a liquid film at the end of solidification and a greater role for elemental segregation in promoting hot-cracking in Cu-containing HEAs. This is consistent with recent work showing that strong Cu segregation during solidification promotes hot-cracking in AlCoCrCuyFeNi alloys, as Cu-rich liquid films facilitate solidification and liquation cracking [35].

### 4.2. Effect of Process Parameters on Density and Processability

In another study, Niu et al. conducted a parameter optimization analysis for CoCrFeNi-HEAs with Mn additions but found no parameter set that completely mitigated hot cracking [20]. They concluded that the alloy is inherently unsuitable for the L-PBF process. Our results align with this conclusion, as we also observed limited processability for CoCrFeNiCu-HEAs produced by L-PBF. This limitation is attributed to rapid solidification, which generates stress concentrations during processing. Moreover, the presence of large columnar grains increases susceptibility to hot cracking due to the rigid strain environment and the presence of long intergranular liquid channels [21]. Both of these characteristics were observed in the present study.

Prior research suggests that fine precipitates or segregations of aluminum at grain boundaries can help suppress cracks in CoCrFeNi-HEAs [22,23]. However, this behavior was not observed in our CoCrFeNiCu-HEA with Cu and Mn additions. Taken together, these observations suggest that hot cracking in L-PBF-processed CoCrFeNiCu-HEAs is driven by rapid solidification, residual stress, segregation of low-melting elements, and the formation of large columnar grains, consistent with previous studies of similar HEAs.

### 4.3. Deformation Behavior

Despite its hot-cracking propensity, the mechanical properties of L-PBF-processed CoCrFeNiCu (UTS: 662 MPa ± 14, YS: 519 MPa ± 29.9, A: 24.74% ± 4.3) are comparable to austenitic stainless steel AISI 316L, with the exception of lower elongation and impact toughness. The reduced ductility is largely due to residual stresses from the L-PBF process, which can be alleviated through stress-relief heat treatments [13,36].

Within this study, the effect of heat treatment on mechanical properties was minimal, likely due to the relatively low treatment temperatures applied. Previous studies by Brif et al. and Lin et al. have investigated the influence of heat treatments on the tensile properties of CoCrFeNi-HEAs. It has been demonstrated that heat treatments at temperatures above 700 °C can effectively improve ductility. However, this improvement comes at the cost of reduced UTS and YS. Lin et al. also examined a heat treatment (500 °C/2 h/FC) similar to the one used in our study [27]. They measured a reduction in residual stresses, but their findings regarding strength and ductility were consistent with the results presented here.

Plastic deformation in FCC materials occurs primarily through mechanisms such as dislocation slip, twinning, and phase transformations. The studied CoCrFeNiCu-HEA exhibits deformation behavior similar to that of other single-phase FCC metals and HEAs [30]. In FCC metals, plastic deformation is primarily governed by dislocation slip, particularly during the early stages of deformation [36,37]. As the load increases and the material enters the plastic regime, dislocations slip along the {111} planes in the ⟨110⟩ direction [38]. This leads to the formation of slip lines, which become visible starting from Load Step 2 in the in situ tensile tests (Figure 8). At the start, slip activity is observed in several grains, particularly at the crack tips, as seen in Figure 8. This activity contributes to work hardening, increasing the material’s resistance to deformation.

As strain increases further, additional slip systems are activated, resulting in the intersection of primary and secondary slip lines (Figure 8 (Step 4)). The cross-slip of dislocations results in changes to grain shape and the formation of sub-grain formations within grains. This process promotes lattice rotation, leading to the localized grain misorientation that is often observed in deformed FCC metals [39].

Interestingly, despite the high levels of stress, no necking was observed at Load Step 4, suggesting that the ultimate tensile strength (UTS) had not yet been reached. This finding is supported by the fact that the maximum stress during in situ testing (σmax = 732 MPa) was significantly higher than that observed during quasi-static tensile tests (UTS = 681 MPa, sample HT3). The higher stress observed during in situ testing may be attributed to strain hardening induced by the repeated interruptions at Load Steps 2, 3, and 4. Each time the tensile test resumed, the material exhibited a higher yield stress, consistent with the Bauschinger effect [40]. This effect occurs due to dislocation rearrangement, where dislocation structures become stabilized, and subsequent reloading requires higher stress to activate dislocation motion.

This study provides insights into the early stages of deformation in the CoCrFeNiCu-HEA, as no necking or ultimate tensile strength (UTS) was reached during in situ tensile testing. The absence of these advanced deformation stages suggests that the observed mechanisms were primarily governed by dislocation slip and slip line formation. Advanced deformation mechanisms, such as TWIP and TRIP, were not observed, likely due to the limited deformation levels achieved during testing.

## 5. Conclusions

In this study, the processability and hot-cracking susceptibility of CoCrFeNiCu powder were investigated using the L-PBF process with a relatively low laser power of up to 250 W. The microstructural evolution and the mechanical properties have been characterized, and the following conclusions are drawn:

(1) Manufacturing of samples with a high density of 99.8% was achieved at a VED of 187.7 J/mm^3^. However, microcracks were also observed. Reducing the volume energy density using lower laser power reduces the occurrence of hot cracks but does not inhibit them in this study.
(2) The observed crack formation mechanism is identified as hot cracking, supported by three key findings:
  - The presence of dendritic structures beneath the crack surface (Figure 5b).
  - Segregation of low-melting-point elements Cu and Mn near the cracks (Figure 5c).
  - Intergranular cracks detected through EBSD measurements, which revealed high residual stress concentrations in particular at high-angle grain boundaries (HAGBs) (Figure 6c).
(3) The in situ tensile tests revealed significant damage evolution within the selected ROIs, including cracks perpendicular to the loading direction, crack networks, and pores. The observations revealed stable crack propagation despite significant microstructural changes, such as slip line formation and increased grain boundary visibility, during elastic to plastic deformation transitions.
(4) Despite the hot-cracking sensitivity, respectable mechanical properties (e.g., ultimate tensile strength of 662 MPa) could be achieved similar to [9] and [13] and superior to as-casted conditions [13].

This study uniquely demonstrates the processability, hot-cracking susceptibility, and in situ mechanical response of CoCrFeNiCu-HEA fabricated by L-PBF, providing new insights into defect evolution and mechanical performance under these specific processing conditions.

Future work will focus on targeted alloying (e.g., Al addition), pre-heating strategies, and optimized scanning to further reduce hot-cracking susceptibility and enhance mechanical performance. This study provides insights into the processability, defect evolution, and mechanical response of L-PBF CoCrFeNiCu-HEA, guiding the development of crack-resistant, additively manufactured HEAs.

## Figures and Tables

**Table 1:** Chemical composition of the metal powder (wt. %).

Co	Cr	Fe	Ni	Cu	Mn	Al, Ti, Zr, V
25.0	22.0	23.27	25.8	2.9	1.2	0.1 each

**Table 2:** Results of Charpy impact tests.

Specimen	Impact Energy [J]	Building Orientation	Temperature [°C]	Condition
AS-RT-V	40	Vertical	20	as-built
AS-RT-H	29	Horizontal	20	as-built
HT-RT-V	31	Vertical	20	400 °C/2.5 h/FC
HT-RT-H	33	Horizontal	20	400 °C/2.5 h/FC
HT-233K-V	34	Vertical	−40	400 °C/2.5 h/FC
HT-233K-H	29	Horizontal	−40	400 °C/2.5 h/FC

**Table 3:** Results of tensile testing.

Specimen No.	YS [MPa]	UTS [MPa]	A [%]	Condition
AS1	468.24	660.61	29.08	As-built
AS2	490.17	658.55	18.91	As-built
AS3	531.09	673.37	26.33	As-built
HT1	539.94	662.42	28.95	Heat treated
HT2	527.99	636.01	12.14	Heat treated
HT3	555.18	680.92	20.41	Heat treated
mean overall	519 ± 29.9	662 ± 14	24.74 ± 4.3	
mean (AS)	496 ± 26	664 ± 6.6	24.8 ± 4.3	
mean (HT)	541 ± 11.1	659 ± 18.4	24.7 ± 4.3	

## Data Availability

The data presented in this study are available on request from the corresponding author.
